# Investigating the time-dependent withdrawal effects of sofosbuvir and/or ribavirin on male mice: a histological and histophotometrical approach

**DOI:** 10.1007/s00210-026-04974-x

**Published:** 2026-02-03

**Authors:** Esraa H. Shahat, Hamza Ahmed El Shabaka, Elham H. A. Ali, Suzan Ahmed, Iman Zakaria

**Affiliations:** 1 Zoology Department, Faculty of Science, Ain Shams University, El-khalifa El- Mamoun St., Abbassia, Cairo, 11566 Egypt; 2 Zoology Department, Faculty of Women for Arts, Science and Education, Ain Shams University, Cairo, 11757 Egypt

**Keywords:** Sofosbuvir, Ribavirin, Testis, Histopathology, Histophotometry

## Abstract

Hepatitis C virus remains a pressing global health issue that has driven extensive research for introducing advanced therapeutic regimens. Among these, the sofosbuvir (SFV)/ribavirin (RBV) drug regimen has had a profound effect on reducing the viral load. Nevertheless, this drug regimen has led to adverse testicular alterations. This study aimed to investigate the restorative effects of SFV/RBV over different durations to determine the possibility of restoring normal histological and histophotometrical testicular tissue. Swiss Albino mice were grouped as total *n* = 80 with subgroups of *n* = 5 per timepoint per group, into control group, SFV group received 41mg/kg once daily of SFV, RBV group administered with RBV 41 mg/kg twice daily, and SFV-RBV group received a combined dose of SFV + RBV, identical to those used separately. All drugs were administered by oral gavage for 5 successive days. Testicular tissue in the SFV, RBV, and SFV-RBV groups, at 5 and 70 days after administration, exhibited pronounced alterations, including spermatocyte degeneration, Leydig cell impairment, and disrupted spermatogenesis. Moreover, altered morphometric parameters were observed, characterized by a reduction in testicular area, a diminished seminiferous diameter, and a regression in spermatic epithelium thickness, which collectively contributed to a decline in the spermatogenic index and the conception index. Conversely, starting from day 140 and persisting through day 175 post-treatment, testicular architecture demonstrated substantial recovery, with restoration of spermatogenesis and improvements across morphometric parameters. The administration of SFV and/or RBV for 5 days induced significant testicular injury, which was reversed after 175 treatments. Restorative effects underscore the potential safety of SFV/RBV regimens in human males.

**Supplementary Information:**

The online version contains supplementary material available at 10.1007/s00210-026-04974-x.

## Introduction

Infection with hepatitis C virus (HCV) represents a global health problem that impacts millions of individuals around the world (Savasi et al. 2010). According to the World Health Organization (WHO), HCV is estimated to affect approximately 50 million people who are living with chronic hepatitis C globally (World Health Organization 2024). Despite the fact that HCV is curable and unpreventable by vaccination, the number of infected people is rising, with an additional 1 million infections recorded annually (Sallam and Khalil 2024). Over the years, the development and integration of antiviral regimens for HCV have evolved substantially, reflecting significant advancements in therapeutic strategies. Conversely, between 2001 and 2011, the preferred therapy for HCV largely relied on the administration of ribavirin (RBV) and pegylated interferon (PEG-IFN). Followed by introduction of first-generation of proteases inhibitors, boceprevir and telaprevir, which represented a critical milestone in shifting the paradigm of HCV treatment strategies (Yau and Yoshida 2014; Irekeola et al. 2022; Di Marco et al. 2025). The landscape of HCV treatment further evolved with the advent of sofosbuvir (SFV), a nucleotide analog inhibitor, which, when combined with RBV, demonstrated potent antiviral activity against all HCV genotypes. The combination of RBV and SFV, direct-acting antivirals (DAAs), advances both safety and efficacy, presenting patients with optimized therapeutic choices (Kashani et al. 2024). DAAs have redefined the standard treatment for HCV, offering cure rates exceeding 95% and dramatically reducing disease-related complications. Meta-analyses confirm that DAAs significantly lower the risk of hepatocellular carcinoma, liver decompensation, and all-cause mortality among treated individuals (Yew et al. 2024). Despite these advances, reviews highlight persistent gaps in treatment uptake and health system integration, which continue to challenge global elimination efforts (Donaldson et al. 2025; Toma et al. 2025).

Moreover, beyond the role of HCV as a leading cause of liver-related mortality, HCV infection is implicated in a spectrum of extrahepatic manifestations. Interestingly, infection exhibits a higher prevalence among males, who consistently demonstrate lower rates of viral clearance compared to their female counterparts, with the potential influence of genetic factors and steroid hormones on sex‑related variation in vulnerability to infectious diseases (Klein 2000; Knapp et al. 2003; Bakr et al. 2006). Moreover, extensive evidence identifies the testis as a primary target organ susceptible to damage induced by both pharmacological interventions and exposure to environmental toxicants (Wong and Cheng 2011; Gao et al. 2015; Soleimanzadeh et al. 2018). These substances decrease testicular function by a variety of pathways, such as changes in spermatogenesis and apoptotic induction (Kong et al. 2025). Previous studies illustrated that concurrent administration of direct-acting antiviral (DAA) agents such as SFV and RBV, administered separately or together, has been associated with sexual dysfunction (Akl and Salah 2020; AboZaid et al. 2021; Ali et al. 2024). This medical condition is characterized by reduced sperm concentration, diminished sperm motility, and disturbances in hormonal profiles, which negatively leads to complications in male fertility (Mahmoud et al. 2024).

Although the impacts of SFV alone, RBV alone, or their combination together have attracted substantial interest, the possibility of reversing their induced testicular damage is unknown. Thus, this study aimed to investigate, over different durations, the possibility of auto restoration of normal reproductive performance and fertility potential by evaluating sperm parameters, histological alternations, histophotometric changes in testicular tissue, and the conception index following treatment with SFV and/or RBV.

## Materials and methods

### Animals

The study utilized 80 (20 for each duration) male Swiss Albino mice (*Mus musculus*), aged 8–10 weeks and weighing 27 ± 3 g. They were sourced from the Theodor Bilharz Research Institute (TBRI). They were maintained in a controlled environment with a temperature of 22 ± 5 °C, relative humidity of 45 ± 5%, and a regulated 12-h light/dark cycle and had ad libitum access to water and food. Mice were fed a balanced standard rodent food pellet (Agriculture-Industrial Integration Company, Giza, Egypt). They were acclimatized for 7 days and maintained on autoclaved, dust-free aspen wood shavings, which were changed twice weekly. The animals were humanely treated in accordance with the guidelines of the National Institutes of Health for the care and use of laboratory animals. The study protocol was officially approved by the Research Ethics Committee of the Faculty of Science at Ain Shams University (Code: ASU-SCI/ZOOL/2022/9/2).

### Drugs

Sofosbuvir (SFV) is a product of Pharco Pharmaceuticals, Alexandria, Egypt, while ribavirin (RBV) is a product of Minapharm, Egypt. SFV and RBV were dissolved in distilled water. Drug calculations were based on the clinical human standard dose and animal equivalent dose (AED). Adaptations and modifications to the data were made following FDA draft guidance originally structured around body surface area considerations (Nair and Jacob 2016).

### Experimental design

The mice groups (*N* = 80) at the beginning of the experiment were divided into four experimental groups. Group 1 (control group) received an equivalent amount of solvent water. Group 2 (SFV group) received 41 mg/kg of SFV orally once daily. Individuals of group 3 (RBV group) received 41 mg/kg of RBV twice daily. Group 4 (SFV-RBV group) was treated orally with a combination of SFV and RBV at the doses mentioned earlier. All doses were administered by oral gavage at a volume of 3 mL/kg body weight per mouse, with adjustments made for any weight loss. Also, all doses were given for 5 successive days, representing a sub-acute exposure period as determined by a preliminary pilot study. Simultaneously, samples were taken from each group for testing each for 35 days for sperm analysis as a primary observatory test until reaching recovery state (5, 35, 70, 105, 140, and 175 days) as a part of pilot study. The trial was then reduced to four durations (5, 70, 140, and 175 days) according to sperm analysis data which were provided at supplementary file. A dedicated approach was taken to minimize the number of animals employed and mitigate their suffering to complete the experimental studies. Each group contained 5 mice per duration.

### Determination of the relative weight of the testis

The body and testicular weights were taken periodically to calculate the relative weight of the testis (g/100 g b.w), which was calculated according to the following equation: relative testis weight (g/100 g b.w) = [(testis weight of animal at the end of the experiment × 100)/(body weight of animal at the end of the experiment)].

### Morpho-functional indicators of the male gonads’ state

At the end of the experiment, diethyl ether anesthesia was applied to the animals prior to sacrifice. Following dissection, the epididymis was carefully removed, and sperm samples were collected for quantitative evaluation. Sperm analysis was performed using light microscopy to determine both the total sperm count and the number of motile spermatozoa (Oliveira et al. 2014; Liu et al. 2021). To assess sperm morphology, a smear on a slide was made and later stained with hematoxylin and eosin. Soon after, the evaluation was performed under a light microscope to characterize the defects of the head and tail (Souza et al. 2021; Zeynali et al. 2025). In total, 200 spermatozoa per sample/individual were evaluated.

### Histological examinations

The testes from the control and treated groups were dissected, cleansed using 0.9% saline, and preserved in an aqueous solution of 4% paraformaldehyde for 24 h for fixation. After that, the tissues were transferred to 70% alcohol and dehydrated in an ascending series of alcohols, 1 h each. The tissue samples were placed in terpineol for 72 h. Followed by, embedding in paraffin wax across three 1-h stages. Eventually, sections approximately 5 µm (µm) thick were cut from the solidified blocks using a Yidi semi-automated microtome (YD-335A semi-automated microtome), and then mounted onto glass slides. Finally, staining was performed with hematoxylin and eosin (HE) for microscopic examination according to the methods of Bancroft and Gamble (2008).

### Digitization of stained testis tissue cross sections

The HE-stained cross sections were digitized and scanned with a slide scanner (LEICA Aperio-LV1, LEICA Biosystem scanner, Wetzlar, Germany). The specific slide-viewing software from the same firm, Aperio ImageScope software (version 12.4.6.5003; Leica Biosystems, Wetzlar, Germany), was used for optimal visualization and detailed analysis of the complete testis cross-sectional architecture. Using a microscope equipped with a camera proved advantageous, as it captured high-resolution images highlighting the staining of the tubules. This technique enabled visualization of images at several magnifications (× 5, × 10, × 20, and × 40) to facilitate testicular measurements. The applied magnification for this technique equals a field of approximately 80–100 intact tubule sections in testicular samples.

### Quantitative histomorphometric-mathematical image analysis methodology

The new mathematical method, which was designed by Sziva et al. (2022), was utilized following sample preparation and HE staining of testicular sections from all experimental groups. This mathematical method was used to demonstrate precise, quantitative histomorphometric and mathematical image analysis measurements. Firstly, the total testicular morphometry measurements were represented by the total testis tissue cross-sectional area. Secondly, the measurements of total seminiferous tubule morphometry were as follows: seminiferous tubule count; total seminiferous tubule number (count) ratio per cross section (CS); and total seminiferous tubule area ratio per cross section (CS). Eventually, single seminiferous tubule morphometry measurements were as follows: average seminiferous tubule diameter, average seminiferous tubule lumen diameter, spermatogenic epithelium area, spermatogenic epithelium area ratio, and average spermatogenic epithelium thickness. The single seminiferous tubule morphometry measurements about ten fields were obtained to be analyzed per animal. Finally, the total interstitial and other tissue area was the last measurement value in this mathematical method.

### Conception index

Conception index was conducted for each experimental group across the selected durations. Five breeding cages were established per group, each containing one male and three virgin, fertile females. Prior to pairing, vaginal smears were performed to confirm that females were in the estrus phase of their reproductive cycle. Conception index was calculated according to the method described by Baek et al. (2022).

### Statistical analysis

The data were expressed as mean ± standard error of mean (SEM). Statistical significance among groups was evaluated using two-way analysis of variance (ANOVA), followed by Tukey test for multiple comparisons among male adult groups, using GraphPad Prism 8.0.2 (San Diego, CA, USA. Furthermore, the statistical significance was defined as follows: *P* < 0.05 (significant), *P* < 0.01 (highly significant), *P* < 0.001 (very highly significant), and *P* < 0.0001 (extremely significant). All statistical analyses were performed with *n* = 5 per group across all timepoints.

## Results

### Relative testes weight

**Fig. 1:**
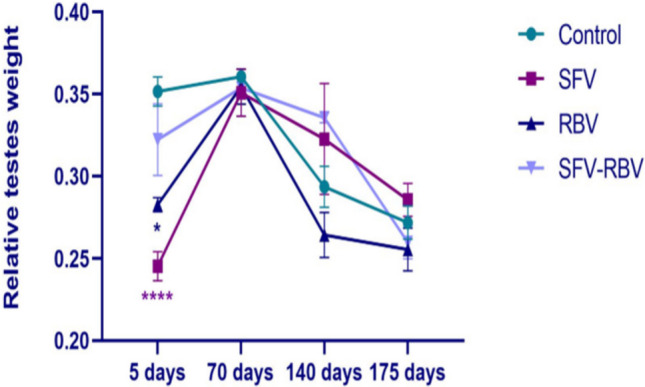
Relative testis weight of the experimental groups across recovery days. Data are presented as the mean ± SEM, with n = 5. *, ****: *P* < 0.05 and *P* < 0.0001 compared with the control group at the same time interval. Statistical analysis was performed by two-way ANOVA followed by Tukey’s

After 5 days of drug administration, although the relative testes weights were significantly decreased in the SFV and RBV groups (0.245 ± 0.009 and 0.283 ± 0.004; *P* < 0.05 and *P* < 0.0001, respectively), there was an insignificant change (*P* ≥ 0.05) in the SFV-RBV group compared with the relevant control group. On the other hand, after 70, 140, and 175 days following treatment with SFV, RBV, and SFV-RBV, the relative testes weights of all treated mice were not significantly changed (*P* ≥ 0.05) compared with the relevant control values (Fig. 1).

### Sperm count and motility

**Fig. 2:**
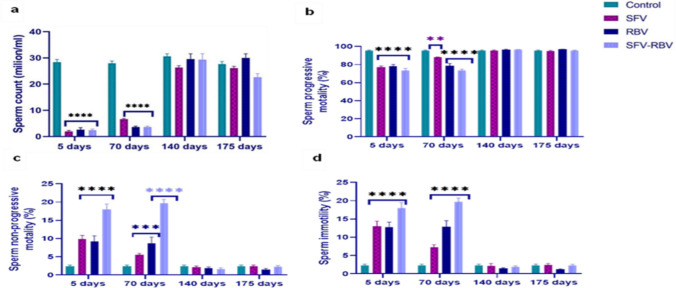
Sperm count (**a**), sperm progressive motility (**b**), sperm non-progressive motility (**c**), and sperm immotility (**d**) of experimental groups along recovery days. Data are represented as the mean ± SEM, with *n*=5.**, ***, ****: *P*< 0.01, *P*< 0.001, *P*< 0.0001 compared with the control group of the same time interval. Statistical analysis was performed by two-way ANOVA followed by Tukey’s multiple comparisons test

After 5 and 70 days post SFV, RBV, and SFV-RBV administration, the sperm count was notably diminished (1.9 ± 0.43, 2.6 ± 0.781, and 2.4 ± 0.51 million/mL; *P* < 0.0001) and (6.6 ± 0.292, 3.6 ± 0.367, and 3.6 ± 0.367 million/mL; *P* < 0.0001), respectively, compared with the relevant control values. However, no significant change (*P* ≥ 0.05) was observed in sperm count at 140 and 175 days post-treatment compared with the relevant control group (Fig. 2a).

The progressive sperm motility percentages of all treated groups were significantly reduced after 5 and 70 days following treatment ((77.09 ± 1.429, 78.14 ± 2.147, and 73.62 ± 2.369%; *P* < 0.0001) and (87.99 ± 0.553 (*P* < 0.01), 78.53 ± 2.73 (*P* < 0.0001), and 73.68 ± 1.38%; (*P* < 0.0001), respectively) versus the relevant control values. However, no significant change (*P* ≥ 0.05) was observed in sperm motility after 140 and 175 days post-treatment compared to the relevant control group (Fig. 2b).

The percentage of non-progressive motility and immotile spermatozoa after 5 days following SFV, RBV, and SFV-RBV administration was significantly elevated ((9.87 ± 0.984, 9.154 ± 1.607, and 17.97 ± 1.509%; *P* < 0.0001) and (13.02 ± 1.41, 12.69 ± 1.45, and 17.97 ± 1.509%; *P* < 0.0001), respectively) compared with the relevant control value. After 70 days following drug administration, the percentage of non-progressive sperm motility of SFV group showed an insignificant change (*P* ≥ 0.05), whereas immotile spermatozoa were significantly elevated (7.306 ± 0.650%; *P* < 0.05). Conversely, both non-progressive motility and immotile spermatozoa were significantly increased in the RBV and SFV-RBV groups ((8.69 ± 1.718 and 19.72 ± 1.036%; *P* < 0.001 and *P* < 0.0001, respectively) and (12.872 ± 1.653 and 19.72 ± 1.036%, respectively, *P* < 0.0001)). However, after 140 and 175 days post-treatment, the percentage of non-progressive motility and immotile spermatozoa was insignificantly changed (*P* ≥ 0.05) across all experimental groups as compared with the respective control group (Fig. 2c and d).

### Sperm morphology

**Fig. 3:**
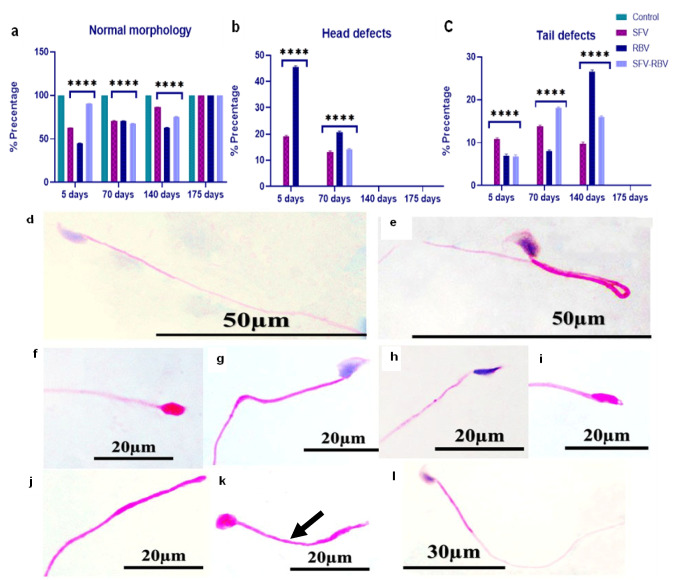
Sperm morphology of experimental groups during the recovery days. **a** Percentage of sperm with normal morphology, **b** percentage of head defects, and **c** percentage of tail defects. **d** Normal sperm and **e** folded sperm. **f**–**j** Head defects: **f** amorphous head; **g** blunt head; **h**, **i** finger head; and **j** without head. **k**,** l** Tail defects: **k** short tail (black arrow) and **l** bent tail. Data are represented as mean ± SEM, with *n* = 5. ****P < 0.0001 compared with the control group of the same time interval. Statistical analysis was performed using two-way ANOVA followed by Tukey’s multiple comparisons test

The percentage of spermatozoa exhibiting normal morphology was significantly decreased following SFV, RBV, and SFV-RBV administration at 5 days (63 ± 0.447, 44.6 ± 0.510, and 90.4 ± 0.400%; *P* < 0.0001), 70 days (71.2 ± 0.374, 71 ± 0.316, and 68 ± 0.316%; *P* < 0.0001), and 140 days (86.4 ± 0.40, 63.2 ± 0.374, and 76 ± 0.447%; *P* < 0.0001), respectively, compared with the corresponding control values. However, at 175 days post-treatment, normal sperm morphology showed no statistically significant difference (*P* ≥ 0.05) in any experimental group compared to the corresponding control (Fig. 3a).

The percentage of sperm head defects 5 days after treatment was significantly increased in SFV and RBV groups (19.2 ± 0.374 and 45.6 ± 0.98%, respectively, *P* < 0.0001) versus their respective control groups. But it was not significantly changed (*P* ≥ 0.05) in SFV-RBV groups at this timepoint. Furthermore, 70 days after treatment, the percentage of sperm head defects was obviously augmented (13.2 ± 0.86, 20.8 ± 0.663 and 14.2 ± 0.374%, respectively, *P* < 0.0001) compared to their respective control groups. However, at 140 and 175 days post-treatment, the sperm head defects showed no significant differences (*P* ≥ 0.05) across all experimental groups compared to the respective controls (Fig. 3b).

The sperm tail defects percentage was significantly elevated following SFV, RBV, and SFV-RBV administration after 5 days (10.8 ± 0.583, 7 ± 0.548, and 6.8 ± 0.583%; *P* < 0.0001), 70 days (13.8 ± 0.86, 8 ± 0.632, and 18 ± 0.548%; *P* < 0.0001), and 140 days (9.8 ± 0.374, 26.6 ± 0.872, and 16.6 ± 0.678%; *P* < 0.0001), respectively. However, by 175 days post-treatment, sperm head defects showed no significant differences (*P* ≥ 0.05) in sperm tail defects observed across any experimental group compared to the respective controls (Fig. 3c).

### Histological studies

**Fig. 4:**
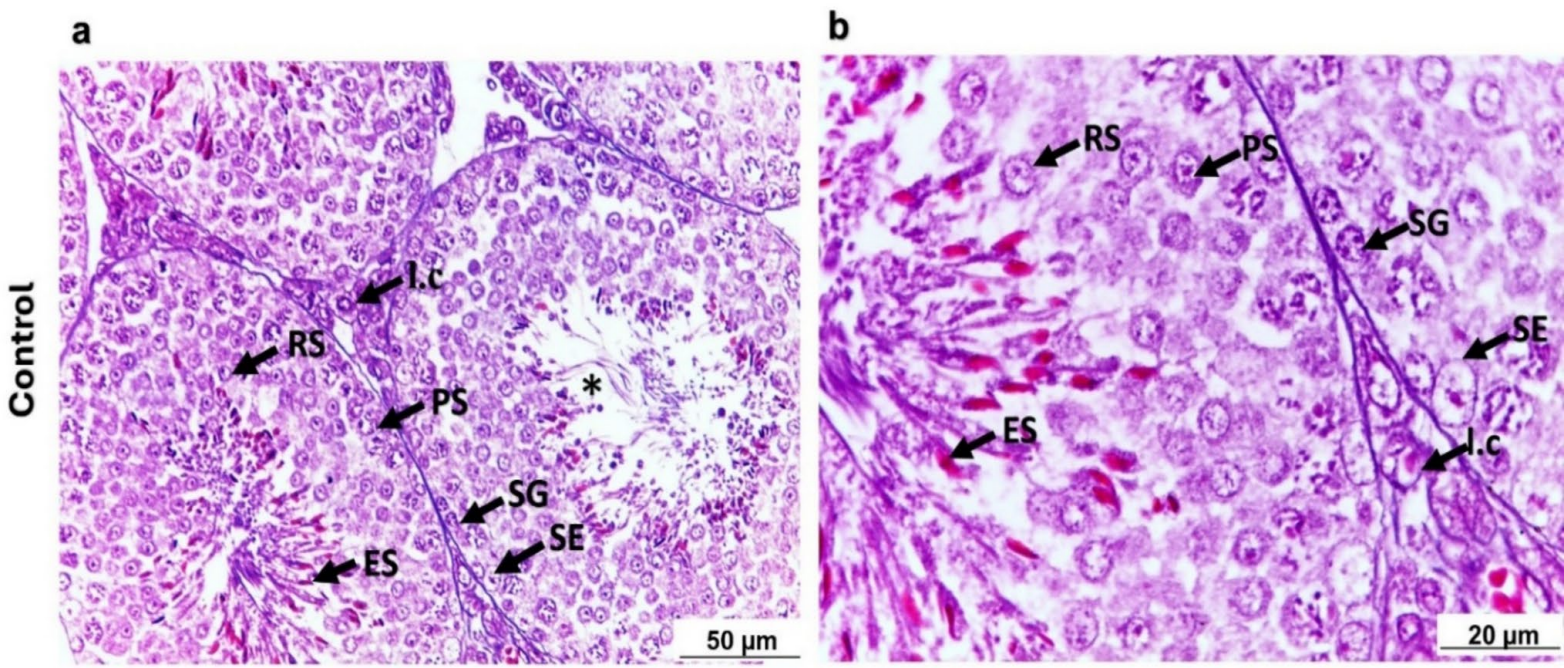
Photomicrograph of H&E-stained testicular sections representative of the normal morphology of mouse testis in control group. **a**: x20 magnification, scale bar = 50 μm; **b**: x40 magnification, scale bar = 20 μm.Control testicular tissue showing seminiferous tubules (asterisk) and interstitial cells (I.c) (Leydig cells). The spermatogonia (SG), primary spermatocytes (PS), rounded spermatids (RS), elongated spermatids (ES) are signs of healthy spermatogenic cells. Sertoli cell (SE) also can be seen

The testis of a control adult mouse is composed of seminiferous tubules with internal structural components and interstitial tissue. Each seminiferous tubule is enclosed by a thin basement membrane consisting mainly of fibroblasts that possess fusiform nuclei. Also, each seminiferous tubule is lined by several germinal epithelial layers situated on the basement membrane and surrounding a central lumen. The germinal epithelium consists of two types of cells, spermatogenic lineage (spermatogonia, primary spermatocytes, secondary spermatocytes, spermatids, and spermatozoa) and somatic Sertoli or supporting cells (Fig. 4).

#### Mice treated with sofosbuvir (SFV), ribavirin (RBV), and a combination dose of sofosbuvir and ribavirin (SFV-RBV) for 5 days of administration

**Fig. 5:**
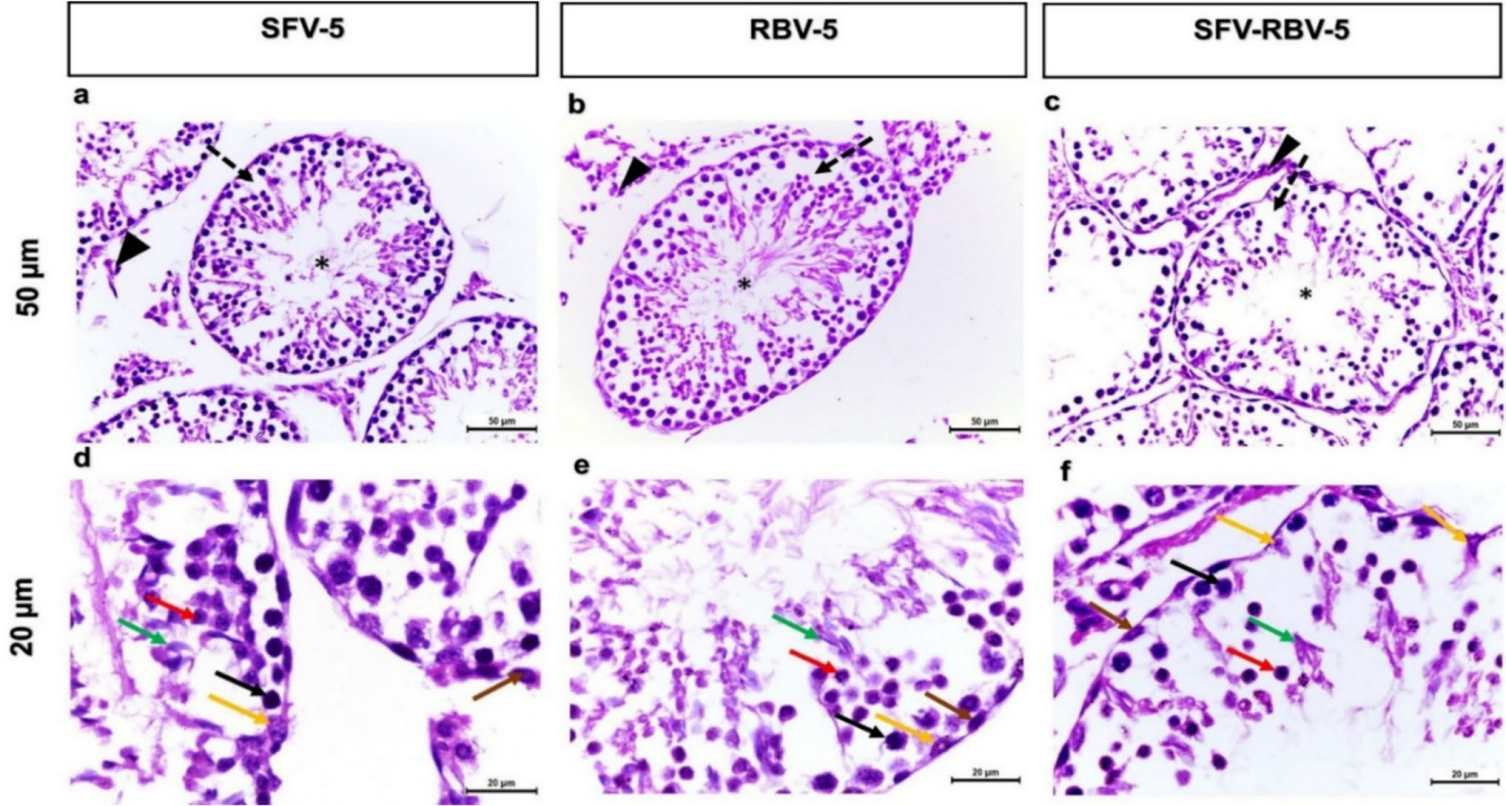
Photomicrograph of H&E-stained testicular tissue sections representative of the histopathology of mouse testis treated with SFV, RBV and SFV-RBV for 5 days. **a–c**: x20 magnification, scale bar = 50 μm; **d–f**: x40 magnification; scale bar = 20 μm. All treated mice showed histological alterations as the seminiferous tubules revealed disarrangement of the spermatogenic cells (dash arrows), reduction of the number of the sperms inside the lumen of the seminiferous tubules (asterisks) and the Leydig cells between seminiferous tubules were degenerated (arrowheads). The signs of pyknosis in the nuclei of the spermatogonia (brown arrows), primary spermatocytes (black arrows), rounded spermatids (red arrows), elongated spermatids (green arrows) and Sertoli cells (yellow arrows) indicated to degenerative spermatogenic cells were noticed at high magnification (**d–f**)

Testis of adult mouse treated with SFV, RBV, and the combination dose of SFV and RBV for 5 days showed common symptoms and impaired seminiferous tubules (Fig. 5). The examination revealed severe cellular degeneration and disorganization in both spermatogenic and interstitial cells. Many spermatocytes and spermatids showed degeneration, and the accumulation of exfoliated germ cells within the lumen tubules was observed. However, seminiferous of testis of adult mouse treated with SFV-RBV for 5 days showed emptiness from germinal cells (Fig. 5a–c). The nucleus of some cells showed various degrees of degeneration as the nuclei of spermatogonia, primary spermatocytes, and spermatids were dark, rounded with signs of pyknosis of SFV and/or RBV groups. Moreover, reduction in the number of the spermatozoa inside the lumen of the seminiferous tubules of all experimental groups was recorded. Besides, Leydig cells between seminiferous tubules of SFV and the combination group of SFV and RBV were degenerated (Fig. 5d–f).

### Mice treated with sofosbuvir (SFV), ribavirin (RBV), and a combination dose of sofosbuvir and ribavirin (SFV- RBV) after 70 days following administration

**Fig. 6:**
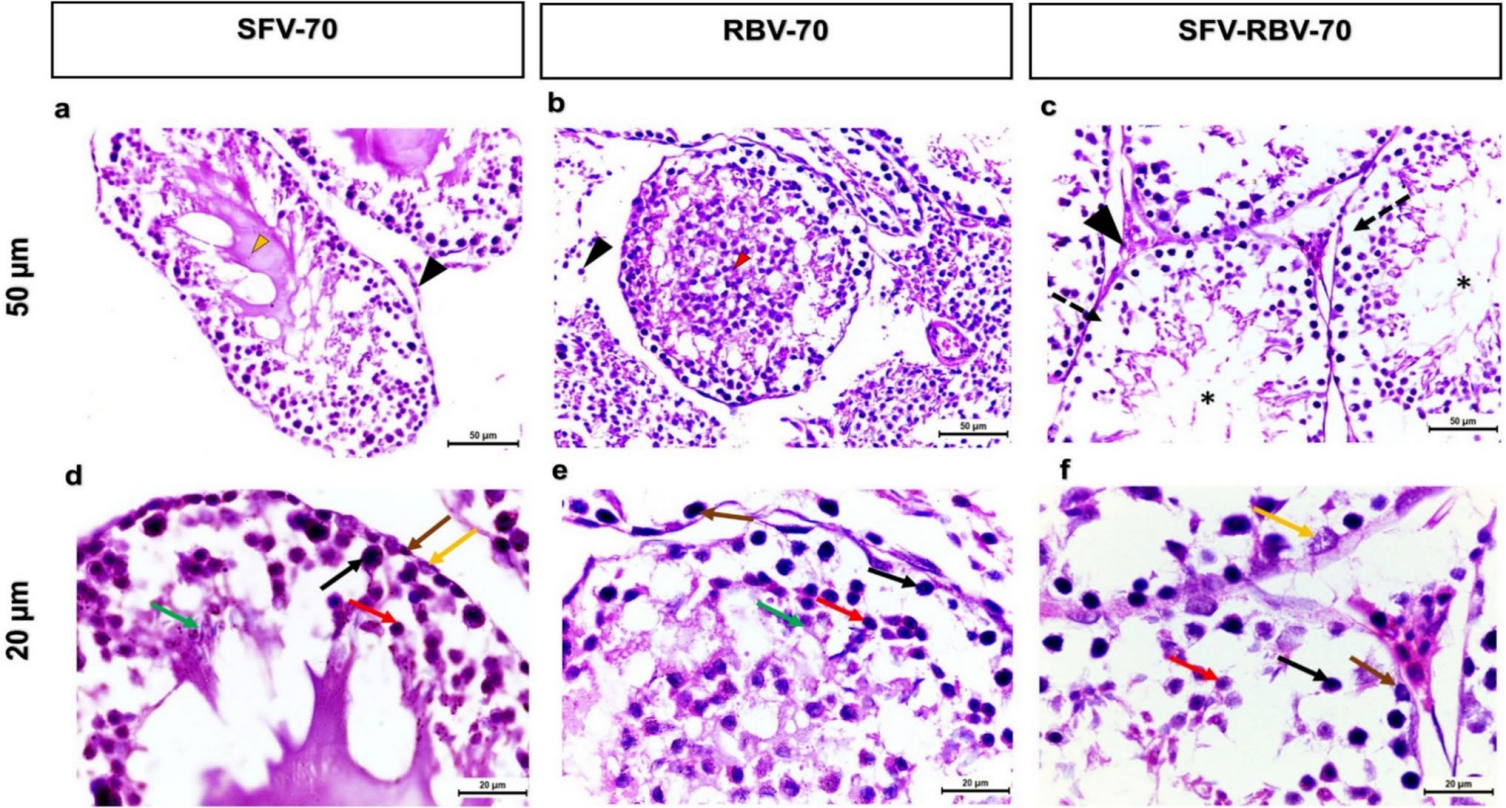
Photomicrograph of H&E-stained testicular sections representative of testicular alterations following 70 days after SFV, RBV and SFV-RBV administration. **a–c**: x20 magnification, scale bar = 50 μm; **d–f**: x40 magnification; scale bar = 20 μm. The seminiferous tubules were noticed with partially necrotic tubules (yellow arrowhead) in group SFV-70 (**a**) and with exfoliation of spermatogenic cells (red arrowhead) in the group RBV-70 (**b**). Besides, vacuolation among spermatic cells (dash arrows) and reduction of the number of the spermatozoa (asterisks) in the lumen of the seminiferous tubules can be seen in group SFV-RBV-70 (**c**). The Leydig cells in all treated groups showed degeneration and reduction in cells number (black arrowheads). The nuclei of the spermatogonia (brown arrows), primary spermatocytes (black arrows), rounded spermatids (red arrows), elongated spermatids (green arrows) and Sertoli cells (yellow arrows) with sings of pyknosis were observed at high magnification (**d–f**)

The testes of adult mice treated with SFV and/or RBV 70 days after administration showed moderately damaged tubules. Also, conspicuous shrinkage and decreased size of the seminiferous tubules, leading to an increase in intratubular spaces, were noticed (Fig. 6a–c). Besides, exfoliation of germ cells was observed (Fig. 6b). Moreover, a reduction in the number of spermatozoa in the lumen of the seminiferous tubules of the SFV, RBV, and SFV-RBV groups was noticed. Additionally, Leydig cells in the SFV and/or RBV groups showed degeneration and a great reduction in cell number (Fig. 6a–c). Furthermore, the general architecture of most of the seminiferous tubules was disorganized with respect to the spermatic lineage. In addition, disarrangements and partial necrosis were observed in most of the seminiferous tubules, especially in the SFV group (Fig. 6d–f).

### Mice treated with sofosbuvir (SFV), ribavirin (RBV), and combination dose of sofosbuvir and ribavirin (SFV-RBV) after 140 days following administration

**Fig. 7:**
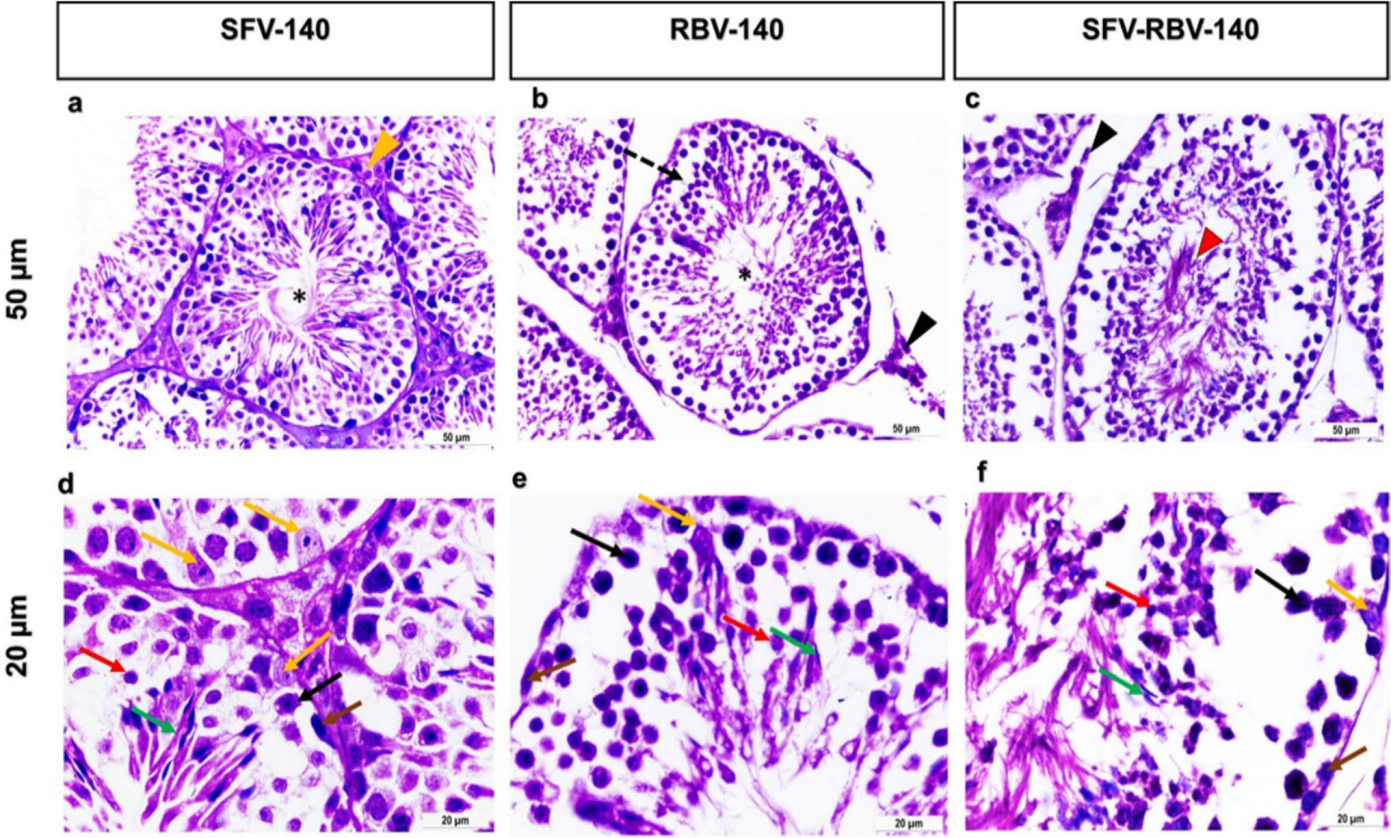
Photomicrograph of H&E-stained sections of testes representative of the testicular histopathology after 140 days following SFV, RBV and SFV-RBV administration. **a–c**: x20 magnification, scale bar = 50 μm; **d–f**: x40 magnification; scale bar = 20 μm. The seminiferous tubules of group SFV-140 (**a**) revealed normal architecture. Moreover, restoration of spermatozoa inside the lumen (asterisks) and normal Leydig cells (yellow arrowhead) were observed (**a,b**). The seminiferous tubules of RBV-140 (**b**) revealed with detachment of the germinal cells lining (dash arrow). Presence of exfoliation of spermatogenic cells (red arrowhead) in group SFVRBV- 140 (**c**). Besides, hypoplasia of Leydig cells (black arrowheads) in groups RBV-140 (**b**) and SFV-RBV-140 (**c**). At higher magnification, the testis of adult mouse of SFV-140 (**d**) showing regular spermatogenesis. Yet, the nuclei of spermatocytes of RBV 140 (**e**) and SFV-RBV 140 (**f**) groups with signs of pyknosis. Spermatogonia (brown arrows), primary spermatocytes (black arrows), rounded spermatids (red arrows), elongated spermatids (green arrows) and Sertoli cells (yellow arrows) (**d–f**)

The testis of adult mouse on the day 140 after treated with SFV showed normal distribution of seminiferous tubules with normal spermatogenesis and renovation of spermatozoa inside the lumen (Fig. 7a and d). Despite the notable improvement and renovation of spermatozoa inside the lumen of the spermatocytes of the testis of adult mouse treated with RBV and the combination group of SFV and RBV, yet disarrangement of the spermatogenic cells and detachment of the germinal cells lining and presence of exfoliation of spermatogenic cells were still observed (Fig. 7b, c, e, and f). The nuclei of some spermatogonia, primary spermatocytes, and spermatids of RBV group exhibited signs of pyknosis (Fig. 7e).

### Mice treated with Sofosbuvir (SFV), ribavirin (RBV), and combination dose of sofosbuvir and ribavirin (SFV- RBV) after 175 days following administration

**Fig. 8:**
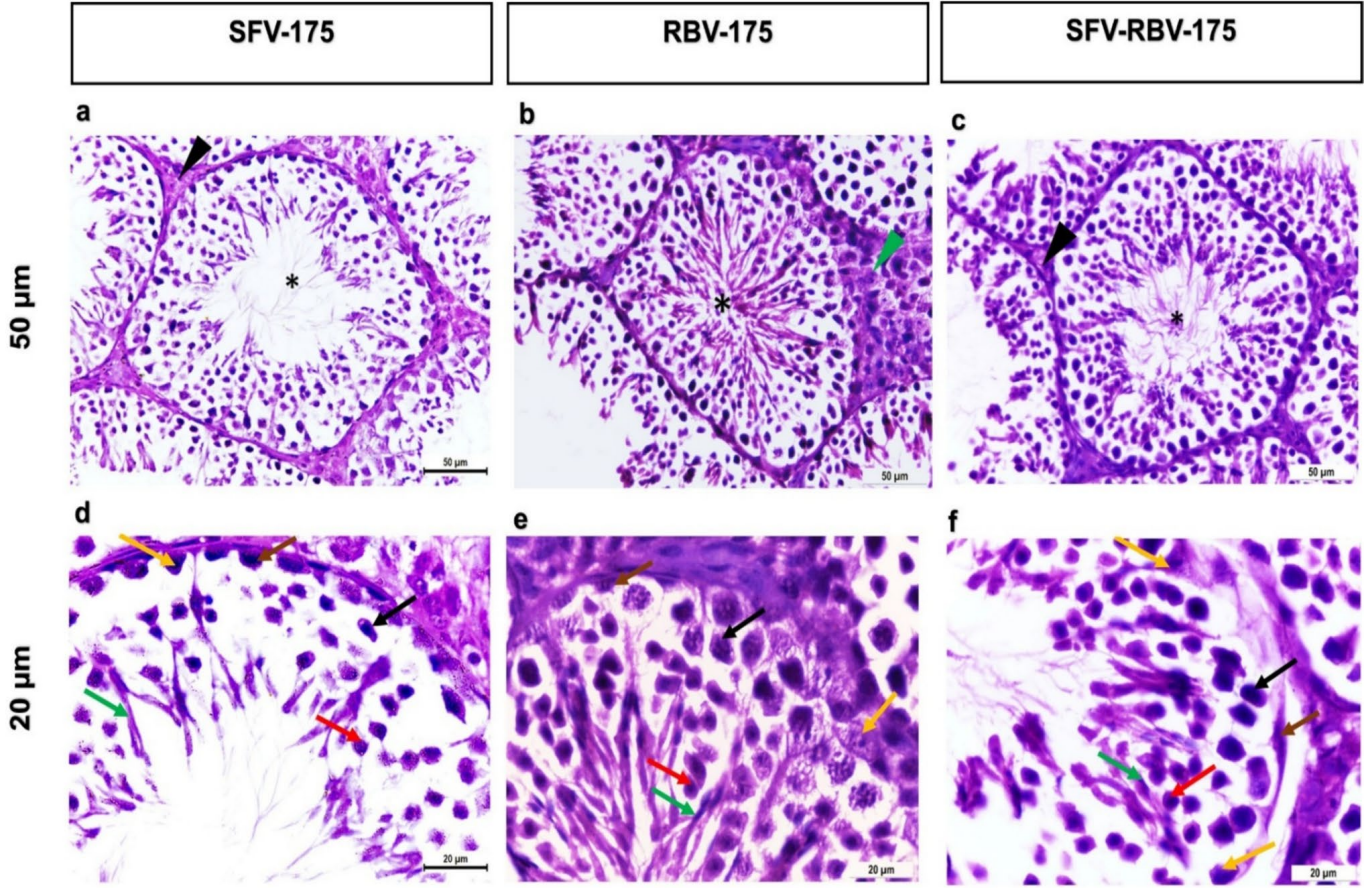
Photomicrograph of H&E-stained sections of testicular tissues representative of the testicular histopathology following 175 days after SFV, RBV and SFV-RBV administration. **a**-**c**: x20 magnification, scale bar = 50 μm; d-f: x40 magnification; scale bar = 20 μm. The seminiferous tubules revealed normal architecture (asterisks), normal distribution of spermatozoa inside the lumen and normal appearance of Leydig cell (black arrowheads) between seminiferous tubules (**a**-**b**). Except of slight increasing of Leydig cells (green arrowhead) in group RBV-175 (**b**). At higher magnification, the seminiferous tubules of groups SFV-175 (**d**), RBV-175 (**e**) and SFV-RBV-175 (**f**) showing regular spermatogenesis with normal appearance the spermatogonia (brown arrows), primary spermatocytes (black arrows), rounded spermatids (red arrows), elongated spermatids (green arrows) and Sertoli cells (yellow arrows)

The seminiferous tubules of testis of adult mouse after 175 days following SFV, RBV and the combination of SFV and RBV administration showed displayed normal distribution of spermatocytes and restoration of spermatozoa inside the lumen. Besides, normal Leydig cells were also observed (Fig. 8a-f). Consequently, the testes of adult mice treated with SFV and/or RBV at the end of treatment period revealed that severe testicular damage and alternations were enhanced along the days and reached the state resembling the normal control tissue.

### Histomorphometrical analysis

#### Total testicular morphometry

**Fig. 9:**
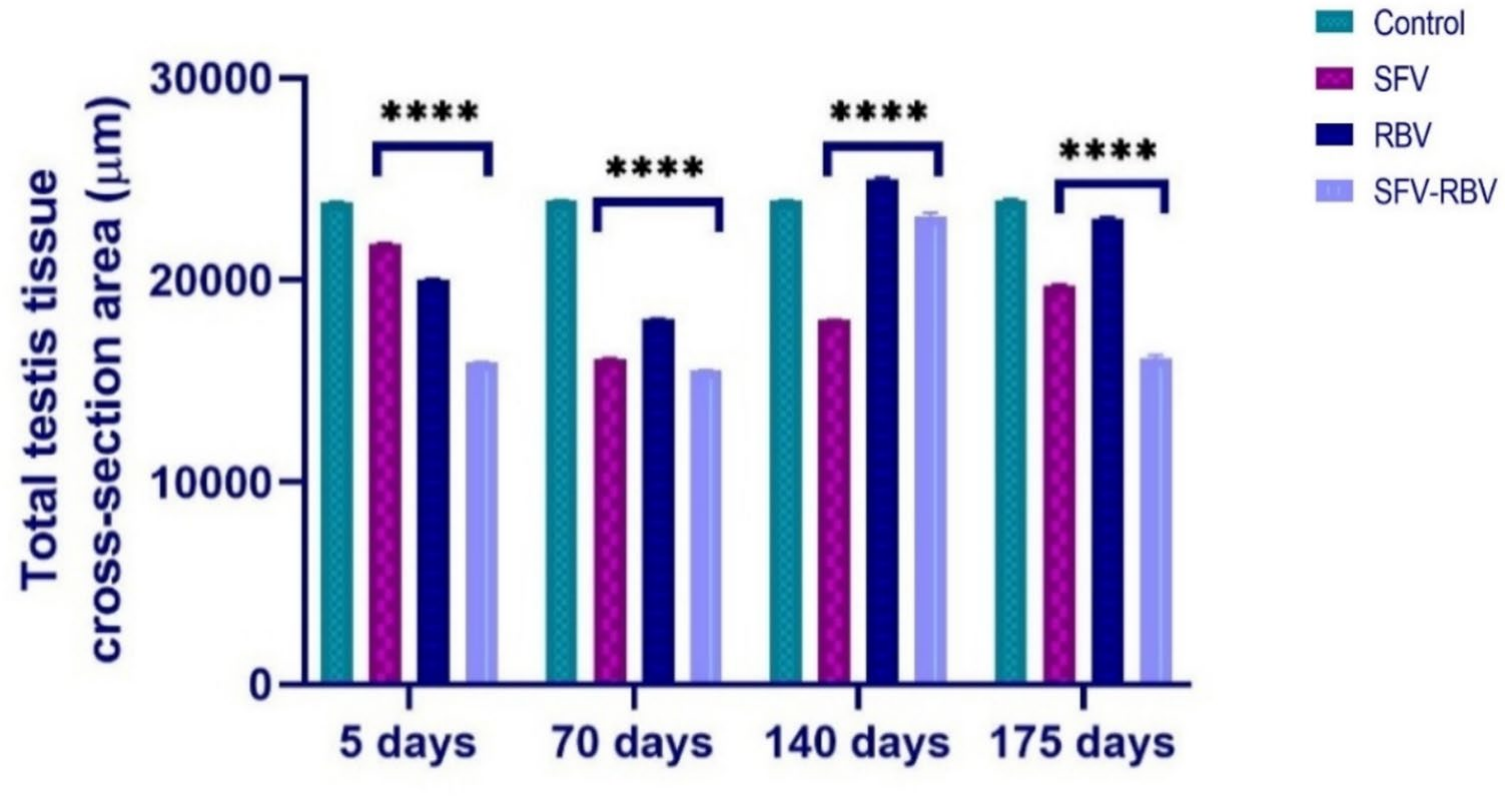
Total testis tissue cross section area of experimental groups along recovery days. Data are expressed as the mean ± SEM, with *n*=5. **** : *p*<0.0001 compared with the control group of the same time interval.Statistical analysis was performed by two-way ANOVA followed by Tukey’s multiple comparisons test

The total testis tissue area cross section after 5 and 70 days following SFV, RBV, and SFV-RBV administration was significantly reduced (21,780.93 ± 60.09, 20,040.79 ± 31.23, and 15,937.00 ± 24.86 µm^2^; *P* < 0.0001) and (16,096.48 ± 31.54, 18,070.87 ± 18.00, and 15,532.49 ± 37.58 µm^2^; *P* < 0.0001), respectively, versus the relevant control values (Fig. 9). At 140 days, the total testicular area remained significantly decreased in the SFV and SFV-RBV groups (18,012.74 ± 28.59 and 23,170.87 ± 165.41 µm^2^; *P* < 0.0001), respectively. Yet, in the RBV group, it was significantly elevated (25,006.13 ± 90.50 µm^2^; *P* < 0.0001) compared to the controls. At 175 days post-treatment, the SFV, RBV, and SFV-RBV groups again exhibited a significant reduction in testicular area (19,737.54 ± 38.12, 23,074.29 ± 54.16, 16,104.15 ± 174.35 µm^2^; *P* < 0.0001), respectively, in comparison with the relevant control group.

### Total seminiferous tubule morphometry

**Fig. 10:**
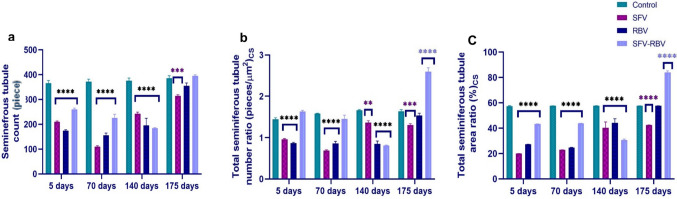
Total seminiferous tubule morphometry of experimental groups along recovery days. (**a**) seminiferous tubule count, (**b**) total seminiferous tubule number (count) ratio cross section (CS), (**c**) total seminiferous tubule area ratio cross section (CS). Data are expressed as the mean ± SEM, with n=5. **, ***, ****: p<0.01, p<0.001 and p<0.0001 compared with the control group of the same time interval. Statistical analysis was performed by two-way ANOVA followed by Tukey’s multiple comparisons test

A significant reduction in seminiferous tubule (ST) count was observed after 5 and 70 days post-SFV, RBV, and SFV-RBV administration ((209.6 ± 3.628, 174.2 ± 3.597, and 259.8 ± 5.257 pieces; *P* < 0.0001) and (110.4 ± 3.172, 156 ± 8.718, and 225.6 ± 14.672 pieces; *P* < 0.0001), respectively) compared with the relevant control group (Fig. 10a). After 140 days post-treatment, ST count remained significantly reduced in all treated groups (242.4 ± 6.608, 196 ± 28.16, and 184.2 ± 2.437 pieces; *P* < 0.0001), respectively. In contrast, by 175 days, a significant decrease in ST count persisted only in the SFV group (314 ± 5.099 pieces; *P* < 0.001), while no significant changes were detected in the RBV and SFV-RBV groups (*P* ≥ 0.05) relative to controls.

The total ST number (count) ratio cross section after 5 and 70 days of drug administration was obviously decreased in the SFV and RBV groups ((0.96 ± 0.016 and 0.869 ± 0.018 pieces/µm^2^, *P* < 0.0001) and (0.68 ± 0.02, 0.863 ± 0.049 pieces/µm^2^, *P* < 0.0001), respectively) compared with the relevant control group. Meanwhile, the ratio in the SFV-RBV group after both durations did not change significantly (*P* ≥ 0.05) compared with the relevant control group. Furthermore, after 140 days following SFV, RBV, and SFV-RBV administration, there was a robust decrease in total ST number ratio cross Section (1.36 ± 0.049 (*P* < 0.01), 0.852 ± 0.068 (*P* < 0.0001) and 0.81 ± 0.007 pieces/µm^2^ (*P* < 0.0001), respectively) compared with the relevant control values. Alternatively, after 175 days following treatment, it had markedly declined in the SFV group (1.30 ± 0.03 pieces/µm^2^; *P* < 0.001). However, that ratio did not change significantly (*P* ≥ 0.05) in the RBV group, while it was significantly augmented (2.59 ± 0.098 pieces/µm^2^; *P* < 0.0001) in the SFV-RBV group relative to the relevant control group.

According to Fig. 10c, the total ST area ratio cross section at 5 and 70 days after SFV, RBV, and SFV-RBV administration was dramatically reduced ((20.01 ± 0.055, 27.31 ± 0.043, and 43.27 ± 0.067%; *P* < 0.0001) and (22.94 ± 0.046, 24.61 ± 0.22, and 43.77 ± 0.109%; *P* < 0.0001), respectively), compared with the relevant control group. In addition, at 140 days after drug administration, it was significantly reduced in the SFV, RBV, and SFV-RBV groups (40.30 ± 4.55, 44.19 ± 3.23, and 30.74 ± 0.612%, respectively; *P* < 0.0001) compared with the relevant control group. At 175 days after drug administration, the total ST area ratio was obviously decreased in the SFV group (42.43 ± 0.082%; *P* < 0.0001). At the same time, it did not change significantly in the RBV group (*P* ≥ 0.05) and it was strikingly exacerbated in the SFV-RBV group (84 ± 1.213%; *P* < 0.0001) compared with the relevant control group.

### Single seminiferous tubule morphometry

**Fig. 11:**
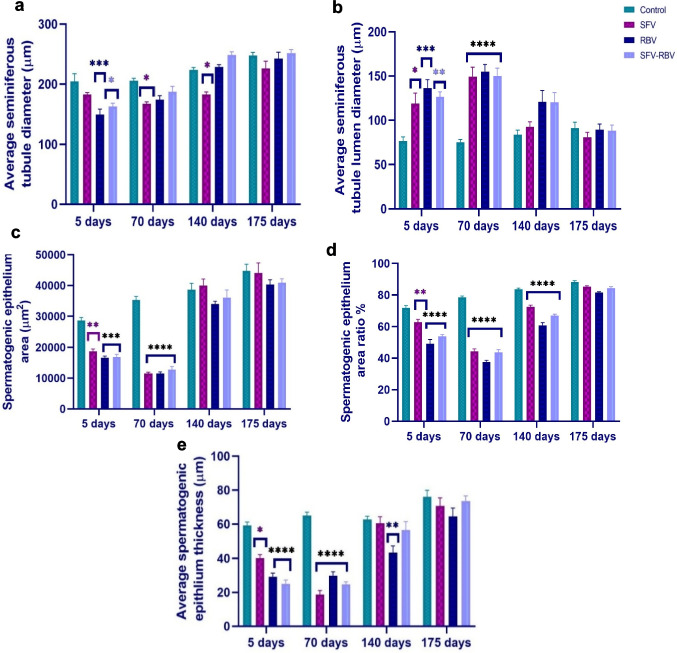
Single seminiferous tubule morphometry of experimental groups along recovery days. (**a**) average seminiferous tubule diameter, (**b**) average seminiferous tubule lumen diameter, (**c**) spermatogenic epithelium area, (**d**) spermatogenic epithelium area ratio, (**e**) average spermatogenic epithelium thickness. Data are expressed as the mean ± SEM, with *n*=5. *, **, ***, ****: *p*<0.05, *p*<0.01, *p*<0.001 and *p*<0.0001 compared with the control group of the same time interval. Statistical analysis was performed by two-way ANOVA followed by Tukey’s multiple comparisons test

Compared with the relevant control group, after 5 days of treatment, there was no notable change (*P* ≥ 0.05) in the average ST diameter in the SFV group (Fig. 11a). Meanwhile, there was a significant reduction in it in the RBV and SFV-RBV groups: 148.92 ± 9.51 µm (*P* < 0.001) and 162.694 ± 5.4 µm (*P* < 0.05), respectively, relative to the relevant control group. Moreover, after 70 and 140 days post-drug administration, although the average diameter of ST had dramatically declined in the SFV group (167.014 ± 3.414 and 182.377 ± 4.58 µm, respectively; *P* < 0.05), it was insignificantly changed in the RBV and SFV-RBV groups versus the relevant control group. Conversely, after 175 days following treatment, the average ST diameter in all treated groups was insignificantly changed (*P* ≥ 0.05) compared with the relevant control group.

According to Fig. 11b, the average ST lumen diameter was strikingly augmented in the SFV, RBV, and SFV-RBV groups at 5 days (118.87 ± 11.89 (*P* < 0.05), 136.03 ± 9.85 (*P* < 0.001), and 126.59 ± 5.51 µm (*P* < 0.01), respectively) and at 70 days following treatment (149.40 ± 10.62, 155.10 ± 8.05 and 150.15 ± 8.77 µm, respectively, *P* < 0.0001) versus the relevant control value. Meanwhile, these changes were substantially recovered by 140 and 175 days post-treatment, as an insignificant difference (*P* ≥ 0.05) was observed in all treated groups versus the relevant control group.

Regarding spermatogenic epithelium (SE), there was a robust reduction after 5 days of SFV, RBV, and SFV-RBV administration in its absolute area (18,679.48 ± 817.45 (*P* < 0.01), 16,634.91 ± 565.43 (*P* < 0.001), and 16,890.76 ± 840.84 µm^2^ (*P* < 0.001), respectively) and in its area ratio (62.81 ± 1.71 (*P* < 0.01), 49.1 ± 2.66 (*P* < 0.0001), and 53.77 ± 1.18 µm^2^ (*P* < 0.0001), respectively) versus the relevant control value. Also, after 70 days post-treatment with SFV, RBV, and SFV-RBV, a prominent decline was observed in SE area (11,467.05 ± 479.95, 11,459.15 ± 559.75, and 12,798.60 ± 959.10 µm^2^, respectively; *P* < 0.0001) and its area ratios (44.37 ± 1.49, 37.52 ± 1.05, and 43.62 ± 1.76 µm^2^, respectively; *P* < 0.0001) versus the relevant control group. Moreover, after 140 days post-treatment, although the area of SE was not significantly changed (*P* ≥ 0.05), its ratio was obviously decreased in SFV, RBV, and SFV-RBV groups (72.48 ± 1.082, 60.62 ± 1.94, and 66.96 ± 0.84 µm^2^, respectively, *P* < 0.0001) compared with the relevant control group. In addition, after 175 days post-drug administration, there was no apparent difference (*P* ≥ 0.05) in both the area and its ratio of SE between all treated groups and the relevant control group (Fig. 11c and d).

Concerning the average thickness of SE (Fig. 11e), there were significant declines in the SFV, RBV, and SFV-RBV groups after 5 days (40.21 ± 1.95 (*P* < 0.01), 29.19 ± 2.11 (*P* < 0.0001), and 24.95 ± 2.24 µm (*P* < 0.0001), respectively) and after 70 days following drug administration (18.65 ± 2.36, 29.89 ± 2.23, and 24.70 ± 1.54 µm, respectively; *P* < 0.0001) versus the relevant control group. However, after 140 and 175 days following treatment, SE thickness showed no significant differences in any treated group (*P* ≥ 0.05), except for the RBV group at 140 days, which exhibited a significant reduction (43.32 ± 3.84 µm; *P* < 0.01) compared to controls.

### Total interstitial and other tissue morphometry

**Fig. 12:**
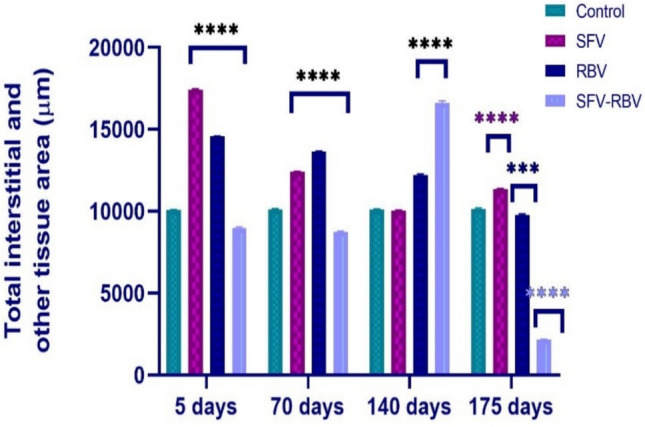
Total interstitial and other tissue area of experimental groups along recovery days. Data are expressed as the mean ± SEM, with *n*=5. ***, **** : *p*<0.001 and *p*<0.0001 compared with the control group of the same time interval. Statistical analysis was performed by two-way ANOVA followed by Tukey’s multiple

The total interstitial and other tissue area 5 and 70 days after treatment was strongly exacerbated in the SFV group (17,422 ± 60.08 and 12,403.4 ± 31.54 µm^2^, respectively; *P* < 0.0001) and in the RBV group (14,567.6 ± 31.22 and 13,653.4 ± 17.97 µm^2^, respectively; *P* < 0.0001) compared with the relevant control group (Fig. 12), whereas it was significantly diminished at the same timepoints in the SFV-RBV group (8979.34 ± 56.54 and 8731.62 ± 37.56 µm^2^, respectively; *P* < 0.001) compared with the relevant control group. Furthermore, 140 days after treatment, no significant change (*P* ≥ 0.05) was observed in the total area of interstitial and other tissue in the SFV group relative to the relevant control group. However, it was significantly elevated in the RBV and SFV-RBV groups (12,206.8 ± 65.64 and 16,612.2 ± 128.26 µm^2^, respectively; *P* < 0.0001) versus the relevant control group. Additionally, 175 days after treatment, it was notably increased in the SFV group (11,360.6 ± 38.12 µm^2^; *P* < 0.0001) but was strikingly decreased in the RBV and SFV-RBV groups (9781.98 ± 54.15 µm^2^; *P* < 0.001 and 2176.9 ± 7.57 µm^2^ *P* < 0.0001, respectively) compared with the relevant control group.

### Conception index

The conception index was completely nullified at 5 and 70 days after SFV, RBV and SFV-RBV administration compared with the control values (Table 1). After 140 and 175 following treatments, SFV, RBV, and SFV-RBV groups had essentially been recovered compared to control values.

**Table 1:** The conception index of experimental groups along recovery days

	C	5 days			70 days			140 days			175 days		
		SFV	RBV	SFV-RBV	SFV	RBV	SFV-RBV	SV	RBV	SFV-RBV	SFV	RBV	SFV-RBV
Conception index (%)^a^	100	0	0	0	0	0	0	87.5	83.3	100	85.7	85.7	100

^a^ $$\frac{\text{number of pregnant females }}{\text{Number of females cohabited}}\times 100$$, *C* control group

## Discussion

The purpose of the present study was to investigate the impact of SFV, RBV, and the combination SFV-RBV administration over different durations (5 days, 70 days, 140 days, and 175 days) on the male reproductive system and functional fertility parameters in adult mice. The results suggest that SFV, RBV, and SFV-RBV administration can increase sperm morphological abnormalities and promote histopathological damage in seminiferous tubules. Besides, it can alter testicular characteristics at morphometric levels and lower the conception index outcomes. Moreover, this study indicates the patination withdrawal timeframe needed to reverse such testicular damage after exposure to SFV, RBV and SFV-RBV treatments.

In this study, the mice, after 5 days of treatment, showed a significant decrease in the relative weight of the testis in the SFV and RBV groups compared with the relevant control group. Previous studies have suggested that administration of antiviral treatment can cause fatigue, loss of appetite, and anemia, which may collectively contribute to weight reduction (Ahmad et al. 2017; Nyström et al. 2019). Furthermore, after 70, 140, and 175 days of drug administration, all experimental groups displayed insignificant changes in the testis relative weight compared with controls, which aligns with the findings of Nkwocha et al. (2022) and Chen et al. (2024). Since, they confirmed that body weight gain is indirectly associated with DAAs treatments after several months of following up compared to the baseline weight.

The functional sperm parameters in the current study after 5 and 70 days of SFV, RBV, and SFV-RBV administration revealed a significant decrease in sperm count and progressive sperm motility. In contrast, a significant increase in non‑progressive sperm motility, sperm immotility, and sperm abnormality was detected at the same time, suggesting sperm maturation dysfunction, which is consistent with Narayana et al.’s (2002) findings following RBV treatment. Yet, no definitive studies on sperm alterations following SFV have been reported. Despite the presence of sperm tail defects at the 140‑day duration in all groups, the rest of the sperm functional parameters by 140 and 175 days following drug administration exhibited an insignificant change, indicating a recovery in sperm functional parameters over time. Contrarily, outcomes reported by Narayana et al. (2005) reveal a reversible effect of DAA detected after only two spermatic cycles, which is approximately 70 days for mice.

Testicular tissue in the current study after 5 and 70 days of drug administration showed notable germ-cell apoptosis and impaired spermatogenesis. The nuclei of spermatogonia, primary spermatocytes, and spermatids were found with signs of pyknosis. Moreover, exfoliation of several seminiferous tubules was detected in the 70-day groups, which is considered a critical hallmark of testicular toxicity and ultimately results in rounded spermatic cells shedding into the tubular lumen (Creasy 2001; Lanning et al. 2002; Creasy et al. 2012), causing testicular dysfunction.

Moreover, conformation of testicular damage associated with SFV and/or RBV administration was demonstrated by Batool and Farzana (2013), as they reported shrinkage of seminiferous tubules as a side effect of RBV injection. Along with the administration of SFV and RBV in a combination dose, extensive distortion and separation of the ad-basal spermatic cells from the underlying basement membrane in most of the testicular seminiferous tubules was reported by El-Kholy et al. (2019). Eventually, germ-cell apoptosis and impaired spermatogenesis led to a reduction in the number of spermatozoa in the lumen of the seminiferous tubules in all the experimental groups, as noticed by Narayana et al. (2002) and Pecou et al. (2009) in previous studies.

However, a tenuous increase in the intertubular spaces was still noted in mice treated with RBV, and a few seminiferous tubules with detached germinal cell linings were noted in the SFV-RBV group after 140 days of drug administration. The seminiferous tubules of adult mice in all experimental groups after 175 days appeared to show continued restoration of the normal distribution of ad-basal and ad-luminal spermatocytes. Also, they exhibited ordinary appearance of spermatozoa inside the lumen at the end of the present study.

In all experimental groups, after 5 and 70 days of drug administration, Leydig cells exhibited degeneration, which led to a remarkable explanation of the detected testicular damage. Leydig cells are considered critical factors responsible for the secretion of androgens, which are essential for sexual maturation, normal spermatogenesis, and the maintenance of reproductive function (Penny et al. 2017). Moreover, these observations may be related to interference with hormonal secretion, resulting in impaired spermatogenesis. By 140 and 175 days, Leydig cells mainly had normal structure and were dispersed throughout the testicular tissue, supporting normal spermatogenesis and suggesting testicular recovery over time, as recorded.

The testicular alternations, particularly within the germinal epithelium, significantly reflect histophotometric parameters in the seminiferous tubules. This diminution likely corresponds to a loss of spermatogenic cell layers and reduced cellular density, as indicative of impaired spermatogenesis. The convergence of histological and sperm functional impairments underscores a direct association between tissue-level damage and compromised testicular performance. Thus, the histophotometric findings serve as an indicator and quantitative correlate of testicular failure in response to SFV and/or RBV administration.

In the current study, the total testis tissue area, ST count, and both ratios following SFV, RBV, and SFV-RBV administration were significantly reduced at all durations, indicating an adverse impact of the drugs. Furthermore, by looking more deeply at single ST morphology, the average ST diameter was not affected as much in any of the experimental groups, regardless of the duration. Except for RBV and SFV-RBV at 5 days and for the SFV group at 70 and 140 days after administration, it was significantly decreased in comparison with the relevant control groups. Such findings could be clarified as follows: testicular dysfunction, which was associated with further accumulation of testicular injury, could emphasize structural changes in the measurements of ST, which could affect sperm concentration.

Afterword the spermatogenic epithelium (SE), the area, area ratio, and thickness of ST at all groups after 5 and 70 days and the SE thickness of RBV following 140 days were significantly decreased in comparison to the relevant control group. Ultimately, the latest results indicate a massive loss in the spermatic cell niche through apoptosis. Additionally, epithelial detachment and exfoliated seminiferous tubules indicated a reduction in the size of the spermatic linage, which disturbed the SE (Owembabazi et al. 2023). The shrinkage of ST, along with the depleted thickness of the germinal epithelium, had been controlled and resulted in a uniform arrangement of spermatocytes with the help of Leydig cell secretions, which are responsible for maintaining SE division (Sampannang et al. 2018; Mäkelä et al. 2019; Anuar et al. 2023). Reflected on, SE area and SE thickness of treated mice after 140 and 175 days of SFV, RBV, and SFV-RBV administration exhibit insignificant change in SE measurements. Such findings conceivably revealed the ability to restore spermatic cells, as shown at the histological level.

On the other hand, the disturbance of spermatic thickness affected the average ST lumen diameter; as by losing the spermatic thickness and impairing spermiogenesis, the tubule lumen diameter becomes wider. Since, the average ST lumen diameter after 5, 70, and 140 days following SFV, RBV, and SFV-RBV administration was significantly increased in comparison to the relevant control group. Ultimately, such findings may refer to the emptiness of the lumen resulting from spermatozoa and spermatocyte sloughing. Except of SFV in 5 and 140 days of drug administration, an insignificant change was observed. Furthermore, 175 days after recovery, the average ST lumen diameter was insignificantly changed in all experimental groups, revealing normal spermatogenesis over time.

The total interstitial and other tissue area of male mice after 5 and 70 days of SFV, RBV, and SFV-RBV administration was significantly increased in comparison to controls as a result of the adverse testicular damage caused by low ST counts, resulting in an increase in the intertubular spaces and affecting Leydig cell proliferation, which led to dysregulation of hormonal secretion. Yet, RBV and SFV-RBV-treated mice showed a significant increase in total interstitial and other tissue area, as they suffered from slight hyperplasia of the interstitial cells after 140 days of administration. Furthermore, after 175 days of recovery, it was significantly decreased in all treated groups, which may lead to a return to the normal state after the elevated level caused by the minimum duration, 140 days, of drug administration.

The DAA metabolism and the mechanism of action are directly associated with oxidative stress and the generation of reactive oxygen species (ROS), a toxicant which subsequently leads to cell death (Kovacic and Somanathan 2015). Toxicant exposure may selectively affect any of the cellular compartments, which may cause germ cell apoptosis and contribute to compromised sperm production (Xie et al. 2014). Yet, long-term toxicant exposure may cause generalized germ cell depletion that represents an advanced lesion, with the potential for testicular atrophy development. Furthermore, testicular atrophy may culminate in a chronic condition of infertility (Boekelheide 2005).

For further confirmation of the results, the conception index was assessed. As in the 5- and 7-day durations, the male mice in all the experimental groups failed to attain mating, resulting in a null conception index. Meanwhile, 140 and 175 days after drug administration, the conception index was enhanced in the SFV, RBV, and SFV-RBV groups, with a prominent enhancement after 175 days following drug administration, indicating successful mating. The present findings are completely consistent with previous studies confirming that disturbances in sperm count, sex hormones, and oxidative stress affect the fertility index. In addition, restoration of the conception index (fertility index) was related to correction of the disturbed factors, which were mostly observed at 140- and 175-day groups (Findeklee et al. 2020; McPherson and Tremellen 2020; Ku et al. 2025).

In conclusion, the extended observation period, combined with comprehensive functional fertility assessments and histological and histophotometric analyses, enhances the predictive value of the current study. The observed restoration of histological integrity and improvement in fertility indices after drug withdrawal indicate a potential for reversibility in SFV-, RBV-, and SFV-RBV-induced testicular damage 175 days after drug administration, highlighting the capacity for endogenous repair mechanisms to mitigate drug-related reproductive toxicity. From a clinical perspective, this study may aid in estimating the timeframe required for the recovery of male reproductive function following antiviral therapy, thereby informing fertility counseling and post-treatment monitoring strategies in patients undergoing SFV and RBV regimens.

## Limitation of the study

While the present study provides valuable insights into the structural and functional consequences of testicular injury, several limitations should be acknowledged. First, the absence of hormonal profiling restricts the ability to fully elucidate the endocrine mechanisms underlying the observed dysfunction. Second, the study was limited to histological and histophotometric assessments without molecular analyses, which may have provided deeper mechanistic understanding. Future studies incorporating hormonal assays, extended time-course evaluations, and multi-modal analyses are warranted to validate and expand upon these findings.

## Supplementary Information

Below is the link to the electronic supplementary material.

ESM 1 (DOCX 35.8 KB)

## Data Availability

The data presented in this study are available upon reasonable request.

## Abbreviations

HCV: Hepatitis C virus
DAA: Direct acting antiviral
SFV: Sofosbuvir
RBV: Ribavirin
ST: Seminiferous tubules
SE: Spermatogenic epithelium
