# Supplementary material for: Investigating the time-dependent withdrawal effects of sofosbuvir and/or ribavirin on male mice: a histological and histophotometrical approach
Source: Naunyn Schmiedebergs Arch Pharmacol. 2026 Feb 3;399(7):10421–37. doi: 10.1007/s00210-026-04974-x (PMC13152939; doi:10.1007/s00210-026-04974-x)
Supplement: Supplementary file 1 — (DOCX 35.8 KB) [file 210_2026_4974_MOESM1_ESM.docx]

**Investigating the Time-Dependent Withdrawal Effects of Sofosbuvir and/or Ribavirin on Male Mice: A Histological and Histophotometrical Approach**

**Journal name: Naunyn-Schmiedeberg's Archives of Pharmacology**

Esraa H. Shahat^1,*^, Hamza Ahmed El Shabaka^1^, Elham H. A. Ali ^2^, Suzan Ahmed^1^

, Iman Zakaria^1^

^1^ Zoology Department, Faculty of Science, Ain Shams University, Cairo, 11566, Egypt.

^2^ Zoology Department, Faculty of Women for Arts, Science and Education, Ain Shams University, Cairo, 11757, Egypt.

^*^Corresponding author. Zoology Department, Faculty of Science, Ain Shams University, El-khalifa El-Mamoun St., Abbassia, Cairo, 11566, Egypt.

E-mail addresses: [esraahshahat@sci.asu.edu.eg](mailto:esraahshahat@sci.asu.edu.eg), [pehshahat@gmail.com](mailto:pehshahat@gmail.com)

**Running title:** Withdrawal effect of Sofosbuvir/ Ribavirin on testicular tissue

**Supplementary Table 1** Spermatozoa count (million/ml) of male mice treated with Sovaldi (SFV), Ribavirin (RBV) and combination dose of Sovaldi and Ribavirin (SFV- RBV) along recovery days.

| Groups  Time  intervals | Control | SFV | RBV | SFV-RBV |
| --- | --- | --- | --- | --- |
| 5 Days | 28.4± 1.030 | 1.9±0.43  ******** | 2.6±0.781  ******** | 2.4±0.51  ******** |
| 35 Days | 28.2± 1.114 | 1.84±0.421  ******** | 2.54±0.771  ******** | 2.26±0.476  ******** |
| 70 Days | 27.9±0.954 | 6.6±0.292  ******** | 3.6±0.367  ******** | 3.6±0.367  ******** |
| 105 Days | 27.6± 1.030 | 6.52±0.267  ******** | 3.46±0.389  ******** | 3.44±0.392  ******** |
| 140 Days | 30.6±0.992 | 26.3±0.752 | 29.5±2.096 | 29.3±2.332 |
| 175 Days | 27.6±1.065 | 26.1±0.696 | 30±1.581 | 22.6±1.373 |

Data represented as the mean ± SEM, (n=5). ********: P < 0.0001 compared with the relevant control group of the same time interval. Statistical analysis was performed by two-way ANOVA followed by Tukey’s multiple comparisons test

**Supplementary Table 2** Percentage of spermatozoa progressive motility (%) of male mice treated with Sovaldi (SFV), Ribavirin (RBV) and combination dose of Sovaldi and Ribavirin (SFV-RBV) along recovery days

| Groups  Time  intervals | Control | SFV | RBV | SFV-RBV |
| --- | --- | --- | --- | --- |
| 5 days | 95.62± 0.522 | 77.098± 1.429  ******** | 78.140± 2.147  ******** | 73.626± 2.369  ******** |
| 35 days | 95.026± 0.538 | 77.2± 1.839  ******** | 77.448± 2.034  ******** | 77.188± 1.956  ******** |
| 70 days | 95.58± 0.557 | 87.99± 0.553  ****** | 78.532± 2.73  ******** | 73.688± 1.381  ******** |
| 105 days | 94.924± 0.485 | 83.92± 1.014  ******** | 77.152± 2.561  ******** | 75.416± 1.344  ******** |
| 140 days | 95.07± 0.578 | 95.656± 0.592 | 96.656± 0.439 | 96.462± 0.438 |
| 175 days | 95.62± 0.687 | 95.118± 0.543 | 96.904± 0.235 | 95.454± 0.570 |

Data represented as the mean ± SEM, (n=5). ******, ********: P < 0.01 and P < 0.0001, respectively compared with the relevant control group of the same time interval. Statistical analysis was performed by two-way ANOVA followed by Tukey’s multiple comparisons test

**Supplementary Table 3** Percentage of spermatozoa non-progressive motility (%) of male mice treated with Sovaldi (SFV), Ribavirin (RBV) and combination dose of Sovaldi and Ribavirin (SFV- RBV) along recovery days.

Data represented as the mean ± SEM, (n=5). *******, ********: P < 0.001 and P < 0.0001, respectively compared with the relevant control group of the same time interval. Statistical analysis was performed by two-way ANOVA followed by Tukey’s multiple comparisons test

| Groups  Time  intervals | Control | SFV | RBV | SFV-RBV |
| --- | --- | --- | --- | --- |
| 5 days | 2.374± 0.391 | 9.872± 0.984  ******** | 9.154± 1.607  ******** | 17.97± 1.509  ******** |
| 35 days | 2.224± 0.301 | 9.372± 0.532  ******** | 9.194± 1.581  ******** | 16.236± 1.607  ******** |
| 70 days | 2.316± 0.377 | 5.526± 0.444 | 8.690± 1.718  ******* | 19.720± 1.036  ******** |
| 105 days | 2.322± 0.354 | 5.868± 0.382 | 8.818± 1.738  ******* | 18.042± 1.174  ******** |
| 140 days | 2.370± 0.395 | 2.156± 0.350 | 1.840± 0.424 | 1.664± 0.356 |
| 175 days | 2.340± 0.316 | 2.378± 0.376 | 1.558± 0.261 | 2.274± 0.398 |

**Supplementary Table 4** Percentage of immotile spermatozoa (%) of male mice treated with Sovaldi (SFV), Ribavirin (RBV) and combination dose of Sovaldi and Ribavirin (SFV- RBV) along recovery days.

Data represented as the mean ± SEM, (n=5). ********: P < 0.0001 compared with the relevant control group of the same time interval. Statistical analysis was performed by two-way ANOVA followed by Tukey’s multiple comparisons test

| Groups  Time  intervals | Control | SFV | RBV | SFV-RBV |
| --- | --- | --- | --- | --- |
| 5 days | 2.343± 0.330 | 13.024± 1.410  ******** | 12.692± 1.459  ******** | 17.97± 1.509  ******** |
| 35 days | 2.299± 0.314 | 12.228± 1.413  ******** | 12.534± 1.169  ******** | 16.156± 1.624  ******** |
| 70 days | 2.264± 0.282 | 7.306± 0.650 | 12.872± 1.653  ******** | 19.720± 1.036  ******** |
| 105 days | 2.294± 0.307 | 6.892± 0.660 | 14.016± 1.607  ******** | 17.704± 1.101  ******** |
| 140 days | 2.323± 0.339 | 2.184± 0.645 | 1.486± 0.187 | 1.870± 0.327 |
| 175 days | 2.295± 0.316 | 2.416± 0.424 | 1.302± 0.075 | 2.258± 0.371 |
